# The relationship of kinesiophobia in patients with lymphedema: a case-control investigation

**DOI:** 10.3389/fpsyt.2024.1293614

**Published:** 2024-02-20

**Authors:** Ana Júlia Monteiro, Carmen de Labra, Marta Elena Losa-Iglesias, Adriano Dias, Ricardo Becerro-de-Bengoa-Vallejo, Helena Silva-Migueis, Filipe Macedo, Daniel López-López, Juan Gómez-Salgado

**Affiliations:** ^1^ Research, Health, and Podiatry Group, Department of Health Sciences, Faculty of Nursing and Podiatry, Universidade da Coruña, Ferrol, Spain; ^2^ Physiotherapy Department, Escola Superior de Saúde da Cruz Vermelha Portuguesa - Lisboa, Lisbon, Portugal; ^3^ Research, Health, and Podiatry Group, Department of Physiotherapy, Medicine and Biomedical Sciences, Faculty of Nursing and Podiatry, Universidade da Coruña, Ferrol, Spain; ^4^ Faculty of Health Sciences, Universidad Rey Juan Carlos, Alcorcon, Spain; ^5^ Epidemiology – Department of Public Health and Grade Program of Public/Collective Health, Botucatu Medical School/Universidade Estadual Paulista (UNESP), Botucatu, Brazil; ^6^ Facultad de Enfermería, Fisioterapia y Podología, Universidad Complutense de Madrid, Madrid, Spain; ^7^ Integrated Continuing Care Unit, Casa de Santa Maria, Camarate, Portugal; ^8^ Department of Sociology, Social Work and Public Health, Faculty of Labour Sciences, University of Huelva, Huelva, Spain; ^9^ Safety and Health Postgraduate Programme, Universidad Espíritu Santo, Guayaquil, Ecuador

**Keywords:** lower limb lymphedema, fear of movement, beliefs, activity avoidance, somatic focus, tampa scale for kinesiophobia

## Abstract

**Introduction:**

Kinesiophobia and lymphedema appear to be related conditions, and it is important to understand this relationship, as many of the symptoms and comorbidities presented by individuals with lower limb lymphedema are prevented and treated through movement, thus constituting kinesiophobia as a barrier to intervention. The objective of this study is, therefore, to evaluate and analyze the kinesiophobic beliefs reported by individuals with and without lower limb lymphedema, regarding the agreement, severity and differences found, and to establish levels of kinesiophobia.

**Methods:**

A case-control study with a total sample of 80 participants (40 with lower limb lymphedema and 40 without) was performed. Both groups (with and without lymphedema) were characterized anthropologically, sociodemographically, and clinically. In the case group, lymphedema was evaluated. Participants in both groups completed the Tampa Scale for Kinesiophobia - 13 items (TSK-13).

**Results:**

Individuals with lower limb lymphedema had higher TSK-13 scores than their matched group without lymphedema. The items belonging to the activity avoidance subscale had the highest agreement and score in both groups. Differences between groups were mainly established for items belonging to the somatic focus subscale, showing that individuals with lower limb lymphedema have kinesiophobic beliefs related to the perceived severity of their lymphedema. The prevalence of kinesiophobia was increased in both groups, but the severity was mild.

**Conclusions:**

Considering the apparent tendency of people with lower limb lymphedema to present kinesiophobia and movement-limiting beliefs regarding the condition, greater attention should be paid to its assessment, prevention and treatment from a multidisciplinary and multimodal perspective, which takes into account the multiplicity of factors inherent to kinesiophobia and lymphedema and thus reduce their impact on the management of lymphedema.

## 1 Introduction

Throughout history, numerous references can be found to the relationship between fear and pain (1). However, it was only in 1983 that Lethem, Slade, Troup, and Bentley introduced a theoretical model that explained the relationship between fear of pain and avoidance of movement or activity (1, 2). The term kinesiophobia was later introduced by Kori, Miller, and Todd, in 1990, as the condition capable of awakening in the person “an excessive, irrational and debilitating fear of movement and physical activity resulting from a feeling of vulnerability to painful injuries or reinjuries” (3). Movement avoidance, in chronic situations due to fear of injury or tremendous suffering, can result in greater physical deconditioning and discomfort (3), more pain, disability (3, 4), and poor quality of life (4). This situation makes the continuation of avoidance and the postponement of returning to domestic and work activities even more likely (3). From the point of view of rehabilitation, kinesiophobia can also be seen as a barrier to adherence to treatment (4), as being able to increase treatment time and decrease patient satisfaction levels (5). More recent studies show a relationship between kinesiophobia and chronic conditions with and without pain (6–14), assuming that the causes of fear of movement may go beyond behavioral inadequacy to the presence of continuous pain (15). Thus, psychological symptoms such as fatigue or exhaustion and fear of physical and/or mental discomfort have also been pointed out as possible causes of kinesiophobia (16).

Lymphedema is a condition that can become chronic (17–20) and, therefore, requires maintenance and monitoring throughout life ( (17, 21)), characterized by being a manifestation of the failure of the lymphatic system and/or lymph transport due to primary causes (primary lymphedema), such as problems in lymphatic development, or secondary causes to another pathological condition (secondary lymphedema) (20–23). Lymphedemas that are not adequately treated are more likely to deteriorate (17, 19). However, managing this condition seems complex, from diagnosis to the choice of therapeutic strategies (22, 24, 25). The reference conservative treatment in the literature is Complex Decongestive Therapy, which integrates manual lymphatic drainage techniques, exercise, compression, and skin care (21). However, untreated or inadequately managed lymphedema can result in the onset or worsening of symptoms and/or comorbidities, such as cellulitis, mobility problems, decreased function of the affected extremity (17), numbness, ulcers (18), cosmetic deformities, loss of strength, reduced range of motion (17, 19), heaviness, musculoskeletal pain, inflammation (17–19) and fatigue (18, 19).

The relationship between lymphedema and kinesiophobia can be found in the literature for the upper (26, 27) and lower limbs (17–19, 28). People with lymphedema can believe that movement of the affected extremity can worsen their clinical condition, decrease the range of motion (19), and increase the severity of the edema (18, 19), so they often avoid its use and exercise. On the other hand, people who develop lower limb lymphedema tend to be more sedentary, which can aggravate some of the symptoms and comorbidities mentioned above, such as fatigue (18).

In the literature, no studies were found completely dedicated to the relationship between lower limb lymphedema and kinesiophobia. No study was also found grading the level of kinesiophobia or analyzing the detailed beliefs or causes that lead to the development of kinesiophobic behaviors, which is relevant to understanding them and presenting strategies to mitigate them. However, four studies (17–19, 28) were found that integrate (among other objectives) the study of this relationship. All studies are case-control studies using a group of individuals with lymphedema and a group of healthy people. The first study relating lower limb lymphedema to kinesiophobia was carried out in a small sample of 17 cases and 18 controls with limitations in describing the characteristics of both groups, in which no statistically significant differences were found between mean TSK scores (17). The remaining studies, two were carried out on individuals with unilateral lymphedema of the lower limbs (18, 19) and one only included individuals with lymphedema secondary to gynecological cancer (28). They all showed statistically significant differences in mean scores between groups, regardless of the outcome measure used to determine kinesiophobia. One of these studies shows that a higher total score and biological and psychological dimensions are more expected in people with lymphedema than in ordinary people (18). These studies show a positive correlation between age, BDI (18, 19), fatigue (18), physical performance, balance (19) pelvic floor symptoms and body image (28), and kinesiophobia.

Taking the literature review into account on the one hand, people with lymphedema of the lower limbs may be more susceptible to the development or worsening of symptoms and comorbidities that are preventable with movement (exercise) and which, on the other hand, can be afraid of movement and that this situation may constitute a barrier to treatment with an increase in the tangible and intangible costs of the disease, we hypothesize that individuals with lower limb lymphedema may present higher scores on the Tampa Scale for Kinesiophobia [13 items] (TSK-13) demonstrating greater fear of moving and kinesiophobic beliefs different from those presented by individuals without the condition. Therefore, our study aims to characterize and analyze the differences in the scores of the Tampa Kinesiophobia Scale-13 Items (total and components), as well as the agreement and score of the items isolates (beliefs) reported by adults with and without lower limb lymphedema, as well as grading your kinesiophobia levels.

## 2 Materials and methods

### 2.1 Design and sample

A case-control analytical observational study was conducted between April 2022 and January 2023, involving people with and without lower limb lymphedema. Individuals with lower limb lymphedema were recruited during a foot health screening performed in an academic clinic in Lisbon (Portugal).

The recruitment of participants in the case and control groups followed a consecutive and non-randomized sampling method, completing a sample of 40 individuals with lower limb lymphedema (cases) and 40 individuals without lower limb lymphedema (controls) matched by age and sex. Inclusion criteria were individuals aged 18 or over, of both sexes, who could consent to participate in the study, with previously diagnosed lower limb lymphedema (case group) or without lower limb lymphedema (control group). On the other hand, exclusion criteria included edema originating from non-lymphatic causes, diagnosis of diseases capable of affecting movement unrelated to lymphedema, and presence of cognitive alterations that would make it impossible to fully or partially understand the study instructions.

This study is reported according to the STROBE criteria (STROBE Statement - Checklist of items that should be included in reports of case-control studies) (29).

### 2.2 Procedure

The data collected in this study were collected through an Office 365 form, by a single senior researcher, following the study protocol. These data were subsequently exported to Excel, thus reducing transcription errors and ensuring the accuracy and integrity of the collection. The data resulting from the self-completion of the Tampa Scale for Kinesiophobia were reported by the participants of both groups (cases and controls), following the same procedure, without the researcher’s intervention.

Baseline measurements were evaluated and included anthropometric and sociodemographic data: age (in years), weight (in kilograms), height (in centimeters), BDI using Quelet’s equation (30), education attainment, professional status, marital relationship, and sex; clinical data: search for other diagnoses, regularity of exercise activity and pain; and lymphedema characterization data: type, the origin/triggering factor, location, staging, and date of diagnosis. These characteristics were used to meet the inclusion and exclusion criteria and better characterize the sample. Except for pain, which seems to present solid evidence, all factors predisposing to or related to kinesiophobia are debatable and not completely consensual. However, factors such as age, BMI, sex, educational level, marital status, and regularity of physical exercise or activity are often analyzed in association with the literature. People of older age (19, 31, 32), higher BMI (13, 33), female (33–35), with lower levels of education (33, 34), without a partner (33), and sedentary (36) are those who show a greater predisposition to develop kinesiophobic behaviors. On the other hand, characteristics of lymphedema such as the cause (primary or secondary lymphedema), location, staging, and duration may also be related to kinesiophobia. Individuals with primary and secondary lymphedema may present different causes of fear of movement, with a higher report of psychosocial causes in individuals with primary lymphedema (18). Differences in limb volume related to asymmetry caused by the uni- and bilateral nature of the condition or the severity of the edema (staging) can also produce symptoms or affect balance (19, 27), which can trigger kinesiophobic behaviors. The duration of the condition/symptoms can also be a major factor, as it can limit physical activity and reduce the biological ability to act (18).

Subsequently, participants from both groups completed a validated measurement instrument: the Portuguese Language Version of the Tampa Scale for Kinesiophobia [13 Items] - TSK-13. The Tampa Scale for Kinesiophobia was originally developed to assess kinesiophobia and assess the level of comfort, safety, and preparation for movement (37). The original scale features 17 items scored on a 4-point Likert scale (1, 37, 38), which allows for the assessment of the subjective perception of safety and confidence with which individuals perform the movement (37). In this version, the final score varies between 17 and 68, indicating that higher scores indicate greater fear of movement (1, 37, 38). Short versions of the Tampa Scale for Kinesiophobia exist and are used, such as TSK-4 (39, 40), TSK-11 (37, 40, 41), and TSK-13 (37, 40, 42). The abbreviated version TSK-13 results from removing reverse items [4, 8, 12, and 16] from the original scale as it appears to improve the psychometric characteristics of the outcome measure (37, 38, 40, 42).

The TSK-13 consists of 13 items scored from 1 to 4 on a Likert scale, which correspond in an increasing way to strongly disagree, somewhat disagree, somewhat agree, and strongly agree. The final score can vary between 13 and 52, with higher scores relating to more severe levels of kinesiophobia. The cutoff points proposed for interpreting the TSK-13 are: subclinical, from 13 to 22 points; mild, from 23 to 32 points; moderate, from 33 to 42 points; and severe, from 43 to 52 points (42). This outcome measure was cross-culturally adapted to Portuguese from Portugal, in 2013, in a clinical sample of individuals with chronic low back pain, showing adequate psychometric properties: good internal consistency (Cronbach alpha of 0.82), exceptional test-retest reliability (ICC of 0.99), good construct validity by correlation with VAS Pain (r=0,691, p<0,001), VAS Confidence (r=-0,772, p<0,001) and duration of the painful episode and low to moderate responsiveness (37).

No analyzes of the psychometric characteristics of any version of the TSK were found in people with lymphedema. TSK is, however, the most used measure outcome in studies that evaluate kinesiophobia in individuals with lower limb lymphedema.

### 2.3 Sample size calculation

For this case-control study, we used OpenEpi 3.01 to determine the necessary sample size based on confidence levels, power, and equal group sizes. We estimated that a minimum of 80 participants (40 per group) would be needed, with a two-sided confidence level of 75% and a power of 80%. The ratio of controls to cases was 1, and the proportion of cases exposed was unknown but estimated to be 50%, while controls were estimated to have a 28% exposure rate.

### 2.4 Ethical considerations

This study was approved by the Ethics Committee of the Portuguese Red Cross Higher Health School of Lisbon (Opinion no. 01/2022), ensuring its procedures’ ethical and legal compliance (43, 44).

### 2.5 Statistical analysis

To compute the statistical analysis, version 29.0 of the Statistical Package for Social Sciences (SPSS) for Windows (IBM Company, Armonk, NY, USA) was used. All analyses established a statistical significance of p<0.05 and a confidence interval (CI) of 95%. Quantitative variables were subjected to descriptive statistics - mean, standard deviation, median, interquartile range, and range (minimum-maximum). The normality of these data was tested using the Kolmogorov-Smirnov Test (45). Differences between the means of the two groups were evaluated using inferential statistics using the Student’s t Test for Independent Samples, when the variables had a normal distribution (46). Faced with a non-normal distribution, the non-parametric correspondence test, the Mann-Whitney U Test, was used to compare the medians of independent samples (47). Categorical variables were also subject to descriptive statistics – frequencies and percentages. To compare frequencies between groups, the Chi-square Test (48) was used.

## 3 Results

### 3.1 Descriptive data

The sample consists of 80 individuals, divided into two groups - cases and controls - each with 40 participants. The sample includes 12 men and 68 women, ranging in age from 19 to 75. **Table 1** displays the anthropometric and sex characterization of the sample. All studied variables showed no statistically significant differences.

**Table 1:** Sample´s anthropometric, sociodemographic characterization.

Descriptive Data		Total Group Mean ± SD Median ± IR Range (min-max) (n = 80)	Cases Mean ± SD Median ± IR Range (min-max) (n = 40)	Controls Mean ± SD Median ± IR Range (min-max) (n = 40)	p-Value
**Age (years)**		51,95 ± 11,60 53 ± 14 (19-75)	51,95 ± 11,67 53 ± 14 (19-75)	51,95 ± 11,67 53 ± 14 (19-75)	1†
**Weight (kg)**		74,02 ± 21,30 69 ± 20 (42-163)	77 ± 26,82 68 ± 30,3 (43-163)	70,20 ± 13,03 70 ± 18 (42-110)	0,736†
**Height (m)**		1,64 ± 0,08 1,65 ± 0,11 (1,48-1,85)	1,63 ± 0,08 1,62 ± 0,12 (1,48-1,78)	1,64 ± 0,07 1,65 ± 0,09 (1,50-1,85)	0,312*
**BMI (kg/m2)**		27,45 ± 7,32 25,25 ± 7,96 (17,26-57,07)	29,11 ± 9,20 26,19 ± 10,41 (19,11-57,07)	25,78 ± 4,28 25,04 ± 6,05 (17,26-36,33)	0,392†
**Sex (N %)**	**Male**	12 (15%)	6 (15%)	6 (15%)	1‡
	**Female**	68 (85%)	34 (85%)	34 (85%)	
**Education Attainment (N %)**	**Mandatory education**	12 (15%)	7 (17,5%)	5 (12,5%)	0,531‡
	**≥ Mandatory education**	63 (85%)	33 (82,5%)	35 (87,5%)	
**Professional Status** **(N %)**	**Professionally active**	64 (80%)	29 (72,5%)	35 (87,5%)	0,094‡
	**Retired**	16 (20%)	11 (27,5%)	5 (12,5%)	
**Marital Relationship** **(N %)**	**Yes**	51 (63,7%)	20 (50%)	31 (77,5%)	**0,011‡**
	**No**	29 (36,3%)	20 (50%)	9 (22,5%)	

In all analyses, p<0.05 was considered statistically significant.

*Student´s T-test.

†Mann-Whitney U test.

‡Chi-Squared test.

SD, standard deviation; IR, interquartile range; BMI, body mass index.

The clinical data of the sample are explained in **Table 2**; were not found statistically significant differences between the groups about the regularity of the exercise. However, the group with lymphedema has a higher prevalence of pain, with statistically significant differences between the groups.

**Table 2:** Sample´s clinical data.

Descriptive Data		Total Group Mean ± SD Median ± IR Range (min-max) (n = 80)	Cases Mean ± SD Median ± IR Range (min-max) (n = 40)	Controls Mean ± SD Median ± IR Range (min-max) (n = 40)	p-Value
**Regular exercise activity** **(N %)**	**Yes**	35 (43,8%)	21 (52,6%)	14 (35%)	0,115‡
	**No**	45 (56,3%)	19 (47,5%)	26 (65%)	
**Pain**	**Yes**	42 (52,5%)	28 (70%)	14 (35%)	**0,002‡**
	**No**	38 (47,5%)	12 (30%)	26 (65%)	

In all analyses, p<0.05 was considered statistically significant.

‡Chi-Squared test.

SD, standard deviation; IR, interquartile range.

The clinical characteristics of individuals with lower limb lymphedema (case group) are summarized in **Table 3**. As can be seen, half of the sample of cases presents primary lymphedema, and the other half is secondary. The prevalence of uni and bilateral lymphedema is also very similar, with 47.5% of cases with only one limb affected and 52.5% with both. Almost half of the sample (47.5%) has had the condition for over ten years. 40% of cases have stage 1 lymphedema.

**Table 3:** Characteristics of individuals with lower limb lymphedema.

Cases N (%) (n = 40)				
**Lymphedema classification**	**Primary**	20 (50%)	**Praecox**	15 (37,5%)
			**Tarda**	5 (12,5%)
	**Secondary**	20 (50%)	**Cancer**	10 (25%)
			**No Cancer**	10 (25%)
**Lymphedema** **location**	**Unilateral**			19 (47,5%)
	**Bilateral**			21 (52,5%)
**Staging of Lymphedema**	**Stage 0**			5 (12,5%)
	**Stage 1**			16 (40%)
	**Stage 2**			8 (20%)
	**Stage 3**			11 (27,5%)
**Lymphedema** **duration**	**<1 year**	1 (2,5%)	**Mean ± SD** **Median ± IR** Range (min-max) *	14,73±13,646 9±23 (0-48)
	**1-5 years**	12 (30%)		
	**5-10 years**	8 (20%)		
	**>10 years**	19 (47,5%)		

*time measured in years.

SD, standard deviation; IR, interquartile range.

### 3.2 Outcome measurements

The relative data for TSK-13 can be found in **Table 4**. Individuals with lymphedema are more in agreement with statements such as “Pain tells me when I should stop doing physical activity, thus preventing it from hurting me” (item 10); “Nobody should have to do physical activity when they feel pain” (item 13); “My body is telling me that I have something seriously wrong” (item 3); and “I’m afraid of accidentally hurting myself” (item 7). Individuals without lymphedema agreed more with items 10, 7, 13, and 8 (“Trying not to make unnecessary movements is the best thing I can do to prevent the pain from worsening”). On the other hand, the item with which the participants least agreed in the case group was item 11 (“It is not safe for a person with my physical condition to be physically active”); in the control group, it was item 5 (“The accident I suffered put my body at risk for the rest of my life”). About agreement, there are statistically significant differences in item 3 (“My body is telling me that I have something seriously wrong”); in item 5 (“The accident I suffered put my body at risk for the rest of my life”); item 12 (“I can’t do everything other people do, because I get hurt very easily”); and item 13 (“Nobody should have to do physical activity when they feel pain”), with the cases group showing, for all, higher agreement.

**Table 4:** Agreement and scoring of the 13 items of the TSK-13, final scores, and components score of individuals with and without lower limb lymphedema.

TSK-13 Items		Total Group n=80	Cases n=40	Controls n=40	p-Value
**Item 1**	**Disagree** N (%)	58 (72,5%)	27 (67,5%)	31 (77,5%)	0,317‡
	**Agree** N (%)	22 (27,5%)	13 (32,5%)	9 (22,5%)	
	Mean ± SD Range (min-max)	1,98 ± 0,914 (1-4)	2,15 ± 0,949 (1-4)	1,80 ± 0,853 (1-4)	0,087*
**Item 2**	**Disagree** N (%)	60 (75%)	27 (67,5%)	33 (82,5%)	0,121‡
	**Agree** N (%)	20 (25%)	13 (32,5%)	7 (17,5%)	
	Mean ± SD Range (min-max)	2,03 ± 0,886 (1-4)	2,27 ± 0,984 (1-4)	1,87 ± 0,757 (1-4)	0,131*
**Item 3**	**Disagree** N (%)	54 (67,5%)	20 (50%)	34 (85%)	**<,001‡**
	**Agree** N (%)	26 (32,5%)	20 (50%)	6 (15%)	
	Mean ± SD Range (min-max)	2,05 ± 0,926 (1-4)	2,45 ± 0,876 (1-4)	1,65 ± 0,802 (1-4)	<,001*
**Item 4**	**Disagree** N (%)	54 (67,5%)	23 (57,5%)	31 (77,5%)	0,056‡
	**Agree** N (%)	26 (32,5%)	17 (47,5%)	9 (22,5%)	
	Mean ± SD Range (min-max)	2,07 ± 0,911 (1-4)	2,35 ± 0,893 (1-4)	1,80 ± 0,853 (1-4)	0,006*
**Item 5**	**Disagree** N (%)	60 (75%)	21 (52,5%)	39 (97,5%)	**<,001‡**
	**Agree** N (%)	20 (25%)	19 (47,5%)	1 (2,5%)	
	Mean ± SD Range (min-max)	1,73 ± 0,95 (1-4)	2,23 ± 1,05 (1-4)	1,23 ± 0,480 (1-3)	<,001*
**Item 6**	**Disagree** N (%)	59 (73,8%)	29 (72,5%)	30 (75%)	0,799‡
	**Agree** N (%)	21 (26,3%)	11 (27,5%)	10 (25%)	
	Mean ± SD Range (min-max)	2,00 ± 0,827 (1-4)	2,07 ± 0,829 (1-4)	1,92 ± 0,829 (1-4)	0,421*
**Item 7**	**Disagree** N (%)	41 (51,2%)	20 (50%)	21 (52,5%)	0,823‡
	**Agree** N (%)	39 (48,8%)	20 (50%)	19 (47,5%)	
	Mean ± SD Range (min-max)	2,40 ± 0,954 (1-4)	2,53 ± 0,905 (1-4)	2,27 ± 0,960 (1-4)	0,235*
**Item 8**	**Disagree** N (%)	50 (62,5%)	21 (52,5%)	29 (72,5%)	0,065‡
	**Agree** N (%)	30 (37,5%)	19 (47,5%)	11 (27,5%)	
	Mean ± SD Range (min-max)	2,19 ± 1,020 (1-4)	2,53 ± 0,987 (1-4)	1,85 ± 0,949 (1-4)	0,003*
**Item 9**	**Disagree** N (%)	56 (70%)	24 (60%)	32 (80%)	0,051‡
	**Agree** N (%)	24 (30%)	16 (40%)	8 (20%)	
	Mean ± SD Range (min-max)	1,99 ± 0,907 (1-4)	2,25 ± 0,899 (1-4)	1,73 ± 0,847 (1-4)	0,009*
**Item 10**	**Disagree** N (%)	33 (41,3%)	15 (37,5%)	18 (45%)	0,496‡
	**Agree** N (%)	47 (58,8%)	25 (62,5%)	22 (55%)	
	Mean ± SD Range (min-max)	2,49 ± 0,871 (1-4)	2,60 ± 0,841 (1-4)	2,38 ± 0,897 (1-4)	0,251*
**Item 11**	**Disagree** N (%)	65 (81,3%)	30 (75%)	35 (87,5%)	0,152‡
	**Agree** N (%)	15 (18,8%)	10 (25%)	5 (12,5%)	
	Mean ± SD Range (min-max)	1,76 ± 0,875 (1-4)	1,90 ± 0,900 (1-4)	1,63 ± 0,838 (1-4)	0,161*
**Item 12**	**Disagree** N (%)	61 (76,3%)	26 (65%)	35 (87,5%)	**0,018‡**
	**Agree** N (%)	19 (23,8%)	14 (35%)	5 (12,5%)	
	Mean ± SD Range (min-max)	1,90 ± 0,880 (1-4)	2,18 ± 0,903 (1-4)	1,63 ± 0,774 (1-4)	0,005*
**Item 13**	**Disagree** N (%)	48 (60%)	19 (47,5%)	29 (72,5%)	**0,022‡**
	**Agree** N (%)	32 (40%)	21 (52,5%)	11 (27,5%)	
	Mean ± SD Range (min-max)	2,21 ± 0,774 (1-4)	2,42 ± 0,747 (1-4)	2,00 ± 0,751 (1-3)	0,013*
**TSK-13 Score**	Mean ± SD Median ± IR Range (min-max)	26,79 ± 7,965 27,50 ± 10 (13-46)	29,83 ± 8,357 29,00 ± 9 (14-46)	23,75 ± 6,303 25,00 ± 10 (13-36)	**<0,001†**

P<0.05 was considered statistically significant.

*Student´s T-test.

†Mann-Whitney U test.

‡Chi-Squared test.

SD, standard deviation; IR, interquartile range; TSK-13, Tampa Scale for Kinesiophobia-13 Items.

About the individual scores of the various items, it was noted that the items with the highest scores, in the cases group, were item 10 (“Pain tells me when I should stop doing physical activity, thus preventing myself from getting hurt”), item 7 (“I’m afraid of accidentally hurting myself”); item 8 (“Trying not to make unnecessary movements is the best thing I can do to prevent the pain from getting worse”). In the control group, items 10 and 7 are joined by item 13 (“Nobody should have to do physical activity when they feel pain”) as the highest-scoring items. The items with the lowest scores are, in the cases group, item 11 (“It is not safe for a person with my physical condition to be physically active”) and the control group, items 11 and 12 (“I can’t do everything that other people do, because I get hurt very easily”). Statistically significant differences were recorded between scores for item 3 (“My body is telling me that I have something seriously wrong”); item 4 (“Other people do not take my health condition seriously”); item 5 (“The accident I suffered put my body at risk for the rest of my life”); item 8 (“Trying not to make unnecessary movements is the best thing I can do to prevent the pain from getting worse”); item 9 (“I wouldn’t feel so much pain if something potentially serious wasn’t going on in my body”); item 12 (“I can’t do everything other people do, because I get hurt very easily”); and item 13 (“Nobody should have to do physical activity when in pain”), with the case group always presenting higher scores.

The levels of kinesiophobia severity for both groups are explained in **Table 5**. Although there were variations in the analysis of each TKS-13 item and its final scores, both groups exhibited mild kinesiophobia severity. However, there were significant statistical differences between the groups regarding the prevalence of subclinical and clinical kinesiophobia cases in individuals with and without lymphedema.

**Table 5:** TSK-13 score interpretation - individuals with and without lower limb lymphedema.

		Total Group N (%) (n = 80)	Cases N (%) (n = 40)	Controls N (%) (n = 40)	p-Value ‡
**TSK-13 Levels of severity**	**Subclinical**	22 (27,5%)	6 (15%)	16 (40%)	**0,012**
	**Clinical**	58 (72,5%)	34 (85%)	24 (60%)	
	(Mild	44 (55%)	23 (57,5%)	21 (52,5%)	
	Moderate	11 (13,8%)	8 (20%)	3 (7,5%)	
	Severe)	3 (3,8%)	3 (7,5%)	0 (0%)	

In all analyses, p<0.05 was considered statistically significant.

‡Chi-Squared test.

TSK-13, Tampa Scale for Kinesiophobia-13 Items.

## 4 Discussion

This investigation aimed to examine and compare the self-reported beliefs of safety and confidence when performing movement tasks using the TSK-13 among adult individuals with and without lower limb lymphedema. Kinesiophobia is a condition associated with chronic illness, whether or not pain is present. There is also evidence to suggest that lower limb lymphedema may be related to fear of movement. Despite this, no published studies have specifically explored differences in how people with and without lower limb lymphedema perceive their fear of movement or have measured it in degrees of severity.

The baseline characteristics of the two groups, cases and controls, were relatively alike, with only two significant differences found: the presence or absence of marital relationship and pain. Based on the literature, both of these variables may relate to kinesiophobia. Marital status may be linked to kinesiophobia (33), with references to having a supportive spouse can be a protective factor (33, 49), as this support and care can reduce fear of the activity and facilitate recovery (33), but this is not universally agreed upon. Other studies have found no significant association between marital status and fear of movement (32, 50). On the other hand, the relationship between pain and kinesiophobia is well documented, with solid evidence of an association between high levels of kinesiophobia and greater pain intensity and a moderate association between high levels of kinesiophobia and high pain severity. Kinesiophobia may predict greater pain severity but not greater pain intensity (4). The explanatory model of this relationship, the fear-avoidance model, advocates that people who experience acute pain may enter a vicious cycle of chronic disability and suffering determined by maladjusted cognitive, emotional, behavioral and functional responses to pain (51).

Our study found statistically significant differences between the final TSK-13 scores between people with and without lymphedema. Can other factors besides the prevalence of pain in the case group account for these differences? As previously mentioned, the relationship between pain and fear of movement has been widely studied, and this symptom may effectively be the determining factor for this difference. However, people with lymphedema may experience signs and symptoms, such as fatigue (18, 52, 53), decreased balance (19, 27, 54), reduced physical performance (17, 19) fear of falling (27, 55), and depression (26, 56–61), and appear in the literature in association with kinesiophobia (9, 18, 19, 26, 27, 31). Movement avoidance is common among people with fatigue regardless of the pathological condition creating it (18 ,62–64). There is even an adaptation of TSK for its evaluation - TSK-Fatigue (65). In the literature there is reference that chronic fatigue can be increased both by avoidance and by excessive physical activity. On the other hand, there is also evidence that exercise is an effective way to deal with fatigue (64). However, dealing with fatigue seems to be dependent on the ability to tolerate the underlying biological phenomenon, so individuals who think they have no control over their illness may demonstrate less ability to deal with fatigue and present greater fear of movement. In individuals with lower limb lymphedema, only one study related to fatigue and fear of movement was found (18). In this study, individuals with lymphedema did not have more fatigue than healthy people, and individuals with primary lymphedema had more fatigue than individuals with secondary lymphedema. This study shows that kinesiophobia can be associated with age, BMI, and fatigue in individuals with primary lymphedema. It is essential to point out that there are statistically significant differences between the groups in terms of age and BMI, which makes it challenging to understand the contribution of each one to kinesiophobia. Balance (9, 27, 66) and physical performance (8, 67, 68) are often related to kinesiophobia in the literature, regardless of the underlying condition associated. In other conditions, in which there are also asymmetries in the volume and weight of a part of the body, it has been shown that these variations can be explanatory of the changes in postural stability found by the change in the center of gravity (69), as well as in other conditions with changes in the somatosensory system, such as pain or pain associated with restricted range of motion, can be precipitating factors for avoiding a task with an adequate level of performance (66). Fear of movement can cause avoidance of physical activity associated with activities of daily living, which can become a vicious cycle that contributes to the worsening of the signs and symptoms of the primary pathological condition (67). Furthermore, kinesiophobia is predictive of the results of lower limb physical performance even with control of pain associated with activity and advancing age is strongly related to increased fear of movement in individuals with reduced physical function (68). In individuals with lower limb lymphedema, only one study established a relationship between balance, physical performance, and kinesiophobia (19). In this study, individuals with lymphedema showed decreased static balance and physical performance compared to healthy individuals. A correlation was found between balance, physical performance, and kinesiophobia in both groups. No studies were found relating fear of falling and depression with fear of movement in individuals with lower limb lymphedema. However, studies carried out in other pathological conditions show that both the fear of falling (9, 10, 27, 67) and depression (13, 31, 39) can be related to kinesiophobia. The coexistence of fear of falling and fear of movement has been demonstrated in people with Parkinson’s disease. In this population, the constructs of the TSK and the Falls Efficacy Scale showed a close correlation, showing that the more harmful the activities are considered, the greater the fear felt (70). On the other hand, the relationship between depression and fear of movement has been indirectly explained by the presence of symptoms or handicaps such as pain (13) or reduced physical function (39). In addition, it should be noted that if the determinants of motor activity are multidimensional, those of motor limitation are also multidimensional, dividing them into biological and psychosocial. Kinesiophobia is one of the most common forms of motor limitation, so it would be too reductive to explain it solely by the presence of pain.

In our study, we found a high prevalence of kinesiophobia in both groups, cases (85%) and controls (60%), albeit with a low degree of severity (mild). No literature was found that established degrees of severity of kinesiophobia in individuals with lower limb lymphedema. Case-control studies carried out in other pathological conditions use the TSK-11 version, which has a different way of grading the level of kinesiophobia than the TSK-13. However, in these studies, high prevalences of kinesiophobia were found in the case groups, regardless of the average age and the level of kinesiophobia was mostly moderate (6, 7). What factors can explain the fear of movement of people without the disease? Could some baseline characteristics be at the origin of these low degrees of kinesiophobia? 35% of individuals without lymphedema included in this study reported pain. As previously mentioned, pain can be a determining factor for kinesiophobia; however, in this case, it cannot explain the 60% of kinesiophobia found in the control group. Factors such as age (33, 68, 71), obesity (33, 34), educational level, and marital status (33) may be associated with fear of movement. However, the most plausible explanation for this finding is related to the multidimensionality of kinesiophobia. Fearful individuals were not necessarily exposed to a traumatic incident (72). The social transmission of fear is a possible phenomenon (73).

Fear is an emotion that prepares the body to face danger. Problems begin when dysfunctions in fear processing trigger psychopathological processes that give rise to phobias, in which fear outweighs the threat or actual risk to which the individual is exposed (73). There are common social beliefs that physical activity/movement/exercise can pose a danger to the integrity of the body. In a study that analyzed beliefs as barriers to the practice of physical activity, fear of injury and apprehension regarding exercise were reported concerns, 32.7% and 35.3%, respectively, in the general population (74). These beliefs could be present in both groups and justify kinesiophobia in the control group. Exercise among individuals with lymphedema has traditionally been considered unsafe (75). This fact may also contribute to the report of fear of movement in the group of cases.

Previous traumatic experiences may also be at the origin of the levels of kinesiophobia presented in both groups. These experiences, due to the brain’s plasticity and adaptability, can trigger rapid responses to threats in the future. Thus, avoidance behaviors or fear of movement can arise from false threat signals that generate inadequate judgments of potential danger. These signals are first perceived and sent to the lateral amygdala and subsequently transmitted to the central and accumbens nucleus passing through the basal nucleus, generating physiological, emotional, and behavioral responses related to fear. The amygdala, as well as other regions of the higher central nervous system, are involved, on the one hand, in the construction of fear, but also in its extinction and learning of safety (76).

No study was found carrying out an individual analysis of kinesiophobic beliefs assessed using the TSK-13. However, in general, it is consensual that a 2-factor model is the one that best explains the variance of kinesiophobia in the TSK-13, resulting in two subscales: Activity avoidance (items 1, 2, 7, 8, 10, 11, and 13) and Somatic focus (items 3, 4, 5, 6 and 9). In this study, the items with the highest agreement and score in both groups mainly belong to the Activity avoidance subscale. Only one of the items (item 3) that showed the highest agreement in the cases group belongs to the somatic focus subscale. On the other hand, the items that recorded statistically significant differences mainly belong to the somatic focus subscale. These findings corroborate the construct of the TSK subscales; that is, the items belonging to the activity avoidance subscale assess beliefs that the activity can cause injury/reinjury or increase in pain, while those of the somatic focus subscale assess beliefs in serious medical problems (77). Thus, individuals with lower limb lymphedema are concerned about their condition and believe it may interfere with their motor capacity. In turn, individuals without the disease can only think that their fear of movement is due to the risks they are exposed to during the activity. However, it is important to note that the somatic focus is characterized by a greater tendency to pay more attention or report physical symptoms, being associated with negative affect, especially anxiety and depression, in women (78). Another study analyzing the typology of depressive symptoms in people with lower limb lymphedema, had already recorded this tendency towards the somatization of symptoms, especially fatigue (61), suggesting that in this specific population health professionals should be particularly attentive to the manifestation of physical symptoms that express, in reality, negative affective states.

An intriguing finding of this study was that, in the group of individuals with lymphedema, the item with the lowest agreement score was item 11 (“It is not safe for a person with my physical condition to be physically active”). What factors could justify this belief? In contrast to other phobias, people with kinesiophobia are generally not aware of the irrationality of their fear, believing that avoidance of movement is actually an appropriate response (51). However, kinesiophobia is not necessarily associated with the perceived safety of physical activity, but rather with the fear or anxiety generated by movement. Taking into account that, in this study, although no statistically significant differences were found between the groups, individuals with lower limb lymphedema reported being more active (52.6%) than the general population (35%). The exercises are indicated in treating and controlling lymphedema (21, 75, 79–81). According to the literature, exercise can improve the affected limb’s range of motion and muscle strength, physical fitness, and quality of life (75), reduce limb volume, and help control BMI (80). Adequate exercise is considered a safe practice that does not exacerbate the signs and symptoms of lymphedema (75, 79). Thus, the belief in the safety of physical activity in these individuals may demonstrate suitability for lymphedema treatment and control strategies. In this context, habituation per se must also be taken into account. Habituation is a non-associative behavior manifested by reduced emotional responses to repeated stimuli. It may be a protective factor, but a deficiency in this process may contribute to the persistence of the phobia (73).

In this study, a sample paired by sex and age was used, which represents strength. Despite this, some limitations must be taken into account in the interpretation and generalization of its results. The reduced sample size makes it difficult to generalize the results. In future studies, a sample size should be considered that allows for more robust conclusions and an analysis of the variables considering the different types, location, stages, and duration of lymphedema. Despite this, a confirmatory analysis of the sample size using the proportion of those exposed in the case (85%) and control (60%) groups showed that a type I error of 10% could have been assumed, that is, less than the initially designed for a 75% confidence level while maintaining 80% power. The randomization of the sample should also be considered in the future. Although consecutive sampling is regarded as the best non-probabilistic method of sample selection, it is a fact that this method can lead to systematic errors related to the methodology for selecting participants and factors that influence their participation (82). The data collection method associated with the type of study can lead to a memory bias since the quality and veracity of the data collected depend on the participant’s ability to remember the facts (83). Although the TSK is the most used outcome measure for assessing kinesiophobia, and the TSK-13 is the most valid, reliable, and responsive short version, it has not been validated in individuals with lymphedema. A study using the TSK to assess kinesiophobia in individuals with lower limb lymphedema reported that participants were confused during the filling, especially when they did not have pain but other symptoms that equally limited movement (17). Thus, the validation of this scale for this specific population or the use of different outcome measures should also be considered in future studies.

## 5 Conclusions

This study suggests that individuals with lower limb lymphedema have higher degrees of kinesiophobia than the general population. Beliefs that movement can cause injury, re-injury, or worsening pain are the most evoked in people with and without lymphedema. However, the beliefs that best distinguish the groups are those related to how medical problems and their severity can limit movement. That said, and taking into account current knowledge (51, 76) and the findings revealed here regarding the kinesiophobic beliefs of individuals with lower limb lymphedema, we advocate the implementation of multimodal and multidisciplinary assessments and approaches that take into account the multiplicity of factors of kinesiophobia, lymphedema and the relationship between the two to reduce their impact on the management of the disease. However, there is an urgent need for more research that helps to understand the multiplicity of factors that condition fear of movement in individuals with lymphedema, randomized controlled trials that determine comprehensive interventions for the problem and that allow the prioritization of its assessment, prevention, and treatment in the Guidelines for lymphedema treatment and rehabilitation.

## Data availability statement

The raw data supporting the conclusions of this article will be made available by the authors, without undue reservation.

## Ethics statement

The studies involving humans were approved by Ethics Committee of the Portuguese Red Cross Higher Health School of Lisbon (Opinion no. 01/2022). The studies were conducted in accordance with the local legislation and institutional requirements. The participants provided their written informed consent to participate in this study.

## Author contributions

AM: Conceptualization, Data curation, Formal analysis, Investigation, Methodology, Supervision, Writing – original draft, Writing – review & editing. Cd: Conceptualization, Formal analysis, Investigation, Methodology, Supervision, Writing – original draft, Writing – review & editing. ML: Conceptualization, Formal analysis, Investigation, Methodology, Supervision, Writing – original draft, Writing – review & editing. AD: Conceptualization, Formal analysis, Investigation, Methodology, Supervision, Writing – original draft, Writing – review & editing. RB: Conceptualization, Formal analysis, Investigation, Methodology, Supervision, Writing – original draft, Writing – review & editing. HS: Conceptualization, Formal analysis, Investigation, Methodology, Supervision, Writing – original draft, Writing – review & editing. FM: Conceptualization, Data curation, Formal analysis, Investigation, Methodology, Supervision, Writing – original draft, Writing – review & editing. DL: Conceptualization, Formal analysis, Investigation, Methodology, Supervision, Writing – original draft, Writing – review & editing. JG: Conceptualization, Formal analysis, Investigation, Methodology, Supervision, Writing – original draft, Writing – review & editing.
